# Culture of mesenchymal stem cells derived from equine synovial membrane in alginate hydrogel microcapsules

**DOI:** 10.1186/s12917-018-1425-0

**Published:** 2018-03-27

**Authors:** Vitor Hugo Santos, João Pedro Hübbe Pfeifer, Jaqueline Brandão de Souza, Betsabéia Heloisa Gentilha Milani, Rogério Antonio de Oliveira, Marjorie Golim Assis, Elenice Deffune, Andrei Moroz, Ana Liz Garcia Alves

**Affiliations:** 1 Department of Veterinary Surgery and Anesthesiology, University of Veterinary Medicine and Animal Science UNESP, District of Rubião Júnior, s / n, Botucatu, São Paulo Brazil; 2 Departament of Statistics, Institute of Biosciences, UNESP, District of Rubião Júnior, s / n, Botucatu, SP Brazil; 3 Departament of Graduate Program in Research and Development: Medical Biotechnology (Professional Master’s) from the Blood Center of UNESP, Blood Centre Division, District of Rubião Júnior, s / n° -, Botucatu, SP Brazil; 4 Departament of Urology, University of Medicine, UNESP, District of Rubião Junior s / n° - Blood Centre Division - Laboratory of Cellular Engineering, Botucatu, SP Brazil; 5 Departament of Bioprocesses and Biotechnology, FCFAR - UNESP, Rodovia Araraquara Jaú, KM 01, São Paulo, Brazil

**Keywords:** Viability, Proliferation, Differentiation, Horses

## Abstract

**Background:**

Mesenchymal stem cells derived from the synovial membrane (MSCSM) have a greater potential for joint regeneration, besides the capacity for chondrogenic differentiation, since they are a source closer to the chondrocytes. This study aimed to cultivate and evaluate viability and differentiation of MSC_SM_ encapsulated in a three-dimensional alginate hydrogel (HA) scaffold. Samples of the synovial membrane of the metatarsophalangeal joint of 4 horses were collected by astroscopic surgery. These were subjected to enzymatic digestion, isolated mesenchymal cells, cultured in monolayers and encapsulated at various concentrations, 10^4^; 20^4^; 50^4^; 10^5^; 20^5^ cells in 1.5% sodium alginate solution. The gelatinization process was carried out and cultured for 4 weeks. Viability and cell proliferation were performed by dissolving the microcapsules and counting with trypan blue. The ratio of live cells and total live cells at intervals 0, 7, 14, 21 and 28 days was analyzed.

**Results:**

For the evaluation of differentiation, histological sections stained with hematoxylin and eosin and toluidine blue were performed. There was no statistical difference in the proportion of live cells between groups over the 28 days. The group of 10^5^ cells obtained a higher total number of living cells at the end of the experiment. Through the histological analysis it was possible to observe at 7 days a low amount of spherical cells with chondrocyte characteristics. On day 21, chondrogenic differentiation became evident, with pericellular and territorial matrix production.

**Conclusions:**

This study demonstrated the efficiency of HA as a scaffold for MSC_SM_ and the chondrogenic differentiation, promising for use in the treatment of joint injuries in horses.

## Background

Osteoarthritis (OA) is one of the main causes of lameness in horses and is associated with poor performance of the equine athlete, physical incapacitation and early withdrawal of the animal from sports activities [1]. Joint cartilage is the main target of degenerative OA changes [2]. Numerous treatment strategies are being developed to improve joint cartilage repair. However, the biological and mechanical properties of the repair tissue formed are inferior to those of native articular cartilage. The difficulty arises because the articular cartilage has limited capacity for self-regeneration [3, 4]. In addition, lymphatic system have been shown to be associated with a reduced amount of blood progenitor cells, limiting the regenerative mechanism [5, 6].

Currently, the therapies are using combined treatments involving mesenchymal stem cells (MSC), biocompatible scaffold and bioactive compounds, as a way of supplying cellular source and mechanical and molecular stimulation, aiming at the morphofunctional restoration of damaged articular cartilage [7, 8]. These factors promote stimuli to improve chondrogenic differentiation [9–11]. Cultures of chondrocytes in alginate beads for 2 weeks, which gave rise to a matrix similar to native articular cartilage, maintaining the phenotype for 8 months, which exemplifies the beneficial action of biocompatible scaffolds in chondrogenic differentiation [12].

The alginate hydrogel is a linear polysaccharide (n-acid gururonic acid-anionic), anionic, capable of reversibly gelatinizing in the presence of calcium or other divalent cations [12–16]. It is widely used in tissue engineering, providing an ideal environment for MSCs, facilitating their spatial distribution, which results in microenvironment that resembles native cartilage in vivo [15, 17–20]. In addition, it has chondroinducing actions to promote the synthesis of components of the specific matrix of cartilage [21–23] which favors the regeneration of damaged cartilage.

To date, most of the published studies concerning chondrogenic differentiation have focused on MSCs isolated from the bone marrow [24, 25]. However, the synovial membrane MSC has attracted considerable attention, since they have a higher chondrogenic potential because it is a more specific cellular source and close to the chondrocytes [26–28]. In animal models, synovial membrane (SM) cells can migrate to articular cartilage defects, where they proliferate and become chondrocytes, producing cartilage-like repair tissue [3, 29]. However, the stimulation conditions need to be better understood to optimize the formation of a fully functional and hyaline articular cartilage.

Considering the above, the objective of this work was to cultivate MSC_SM_ encapsulated in alginate hydrogel in different concentrations, comparing the viability, proliferation and chondrogenic differentiation, for posterior use in implants aiming the regeneration of the articular cartilage of horses. Thus, the hypothesis is that alginate microcapsules containing large number of MSC_SM_ cells (100 thousand cells) retain cell viability and chondrogenic differentiation, and their local administration into the articular cavity may contribute to effective intra-articular treatment of osteoarthritis in horses.

## Methods

### Synovial membrane (SM) collection and culture

The synovial membranes were collected from arthroscopies performed in horses attended by the Department of Large Animals Surgery of the State University Julio de Mesquita Filho (UNESP), Campus Botucatu / SP, from April to September, 2016, obtaining it with the written consent of the owner to use the animal in its study.

As synovial membrane donors, four horses were used, two males and two females, with a mean age of 4 ± 0.40 years, weighing on average 400 ± 5.77 kg, presenting joint diseases (osteoarthritis). The anesthetic protocol will be composed of: acepromazine (0.05 mg / kg, IM) and xylazine (0.5 mg / kg, IV) as preanesthetic medication, diazepam (0.15 mg / kg, IV) and ketamine (2.2 mg / kg, IV) for induction and maintenance in the surgical plane with inhalation anesthesia with isofluorane vaporized in 100% oxygen [7]. Of these animals, fragments of synovial membrane (SM) of the right and left metatarsophalangeal joints were obtained with the help of the Rongeur Ferris Smith tweezers. It is important to point out that these animals were only donors of the synovial membrane, since the experiment used allogeneic cells.

The sample obtained from each collection was submitted to successive washes with DMEM Knockout®, followed by mechanical separation by scalpel blade and digestive action with collagenase type I solution (2 mg / mL) diluted in DMEM (Dulbecco’s Modified Eagle’s Medium) Knockout® medium. The solution was homogenized at 37 °C and 5.0% CO_2_ overnight, and after that time the same volume of DMEM Knockout medium was added with 10% heated fetal bovine serum (FBS). This material was centrifuged at 628G for 10 min, the supernatant was removed and the culture medium was added for further centrifugation. Again the supernatant was removed and culture medium was added. MSC_SM_ were cultured in 75cm^2^ culture flasks at a concentration of 10 × 10^4^ cells/cm^2^ using Knockout® DMEM culture medium with 10% FBS. The flasks were kept in an environment controlled oven at 37 °C and 5.0% CO_2_.

### Microcapsules of alginate

Cells at the end of the monolayer culture were encapsulated at various concentrations: 10^4^; 20^4^; 50^4^; 10^5^; 20^5^ cells per microcapsules in a 1.5% sodium alginate solution at pH 7.4. The alginate cell suspension was placed in a 10 mL syringe with a 21 G needle, and was then dispensed from the syringe by dripping into the gelatinization solution (CaCl 2 - 102 mM), thereby allowing the alginate polymerization for 10 min until forming the “hydrogels”. The gelatinization solution was discarded, and the microcapsules were washed 3 times in 5 vol. 0.15 M NaCl [30]. The microcapsules were cultured in Knockout™ DMEM medium. The culture was maintained in a 37 °C oven in a humid atmosphere at 5% CO_2_ and 95% air for 4 weeks. The culture medium was changed every 2 days.

### Cell viability

The recovery of the hydrogel cells to assess cell viability was performed by dissolving the hydrogel in sodium citrate 4% for 20 min in a 37 °C oven. Subsequently the sample was centrifuged and diluted in the solution of trypan blue 0,4%. Cell counting and determination of cell viability was performed using the Neubauer chamber at 5 intervals, described below: intervals 0 (post-encapsulation), intervals 1 (7 days), intervals 2 (14 days), intervals 3 (21 days) and intervals 4 (28 days), using 10 microcapsules in each evaluation. The number of live cells was determined by the exclusion technique of killed cells stained with trypan blue solution.

### Cellular morphology and characterization of the extracellular matrix

In order to evaluate the production of extracellular matrix components the samples were evaluated in two intervals: 7 days and 21 days after encapsulation. The samples were fixed in 10% formaldehyde, passed through increasing solutions of alcohol (70%, 95% and 100%), embedded in paraffin and later sectioned in the microtome. Sections obtained were stained with 0.3% toluidine blue (TB), pH 3.65 and Hematoxylin and Eosin (HE).

### Immunophenotypic characterization - flow cytometry

The progenitor cells were differentiated into the adipogenic, osteogenic and chondrogenic strains demonstrating their multipotentiality. All the differentiations were carried out in triplicate for each animal, and an additional sample per animal was maintained in a basal culture medium for 14 days (as control of adipogenic and osteogenic differentiation) and for 21 days (as a control of chondrogenic differentiation).

For adipogenic differentiation, cells from the third passage were incubated at a density of 20,000 cells/cm^2^ in a 24-well culture dish and cultured in adipogenic culture medium for 14 days. The culture medium (STEMPRO® Adipogenesis Differentiation Kit, Gibco, Grand Island, NY, USA) was changed every 3 days. Subsequently the cells were fixed with 10% formoldehyde solution for 10 min, followed by PBS washes being stained with Oil Red O (Gibco, Grand Island, NY, USA).

To perform the osteogenic characterization of MSC_SM_, the third passage progenitor cells were incubated at a density of 20x10^3^cells/cm^2^ in culture plate 24 well and maintained in osteogenic conditions for 14 days, the medium (STEMPRO®, Osteogenesis Differentiation Kit, Gibco, Grand Island, NY, USA) was changed every 3 days. Cells were fixed in 10% formaldehyde solution for 10 min, followed by sterile water washes being stained with Alizarin Red (Gibco, Grand Island, NY, USA).

Chondrogenesis was induced in a micromass pellet prepared with 1 × 10^6^ cells allocated in a 15 ml conical tube of polypropylene. The pellet was cultured at 37 °C with 5% CO_2_ in 2 mL chondrogenic culture medium (STEMPRO®, Chondrogenesis Differentiation Kit, Gibco, Grand Island, NY, USA), the medium was changed every 3 days. After the 3 week incubation period, the pellet was fixed in 10% formaldehyde solution for 24 h at room temperature, this was paraffin and subsequently cut into sections of 5 μm, stained with hematoxylin for general histology and with Alcian blue to detect the sulfated proteoglycans.

**Fig. 1:**
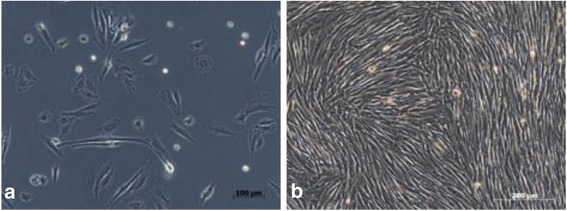
Phase contrast inverted microscopy of the mesenchymal stem cell culture derived from the equine synovial membrane, in monolayers. **a** Cell culture after 36 h of culture, adhere cells with objective 20X, fibroblastoid morphology; **b** Monolayer cell culture, confluent after 2 weeks in culture, objective of 20X

The criteria for characterization of MSCs from horses are based on a marker panel [31] and include several of the criteria that are used to characterize human MSCs, as determined by the International Society for Cellular Therapy [32]. The selection of the antibodies was partially based on a previous study about the knowledge of the researches in equine MSCs [22]. Progenitor cells should express CD29, CD44, and CD90 markers and not express the CD14, CD79, and MHC-II markers. Flow cytometry was performed on the first and third pass, FACS Calibur (BD, San Jose, CA, USA), using forward scatter versus side scatter, evaluating all cells in the sample, with the antibody mouse anti-mouse CD90-FITC monoclonal antibody (Caltag Laboratories, Burlingame, CA, USA) and mouse anti human CD105-FITC mAb (AbD Serotec, Kidlington, Oxford, UK) to evaluate the expression interspecies. The monoclonal mouse anti-mouse CD44 (AbD Serotec, Kidlington, Oxford, UK) and MHC Class II anti-horse mouse (AbD Serotec, Kidlington, Oxford, UK) were labeled with the monoclonal goat anti-mouse IgG -FITC (Molecular Probes, Eugene, OR, USA) (Fig. 1).

### Statistical analysis

The data collected in triplicate on the number of live and dead cells observed in 10 microcapsules at different concentrations (10^4^; 20^4^; 50^4^; 10^5^; 20^5^ cells) and on different days (0, 7, 14, 21 and 28) were analysis using a generalized linear model for Binomial distribution with logistic link function. In order to analyze the total cell count at different concentrations (10^4^; 20^4^; 50^4^; 10^5^; 20^5^ cells) and on different days (0, 7, 14, 21 and 28), a generalized linear model was used for the Negative Binomial distribution Because the data presented high variability. The statistical differences observed for the concentrations and days were tested, being considered statistically significant differences when the *p*-value of the test was less than 5% (*p* < 0.05). Statistical analysis was performed using SAS software version 9.3 (2011).

## Results

### The ability of MSC_SM_ to differentiate

The cell colonies of the present study showed adherence to the plastic in the first 36 h of culture (Fig. 1a). At 15 days cell colonies were similar to fibroblasts in all cultures (Fig. 1b). Cell culture was maintained until the third passage using samples from four animals. The ability of MSC_SM_ to differentiate was confirmed using commercially available means of differentiation (Gibco, Grand Island, NY, USA).

**Fig. 2:**
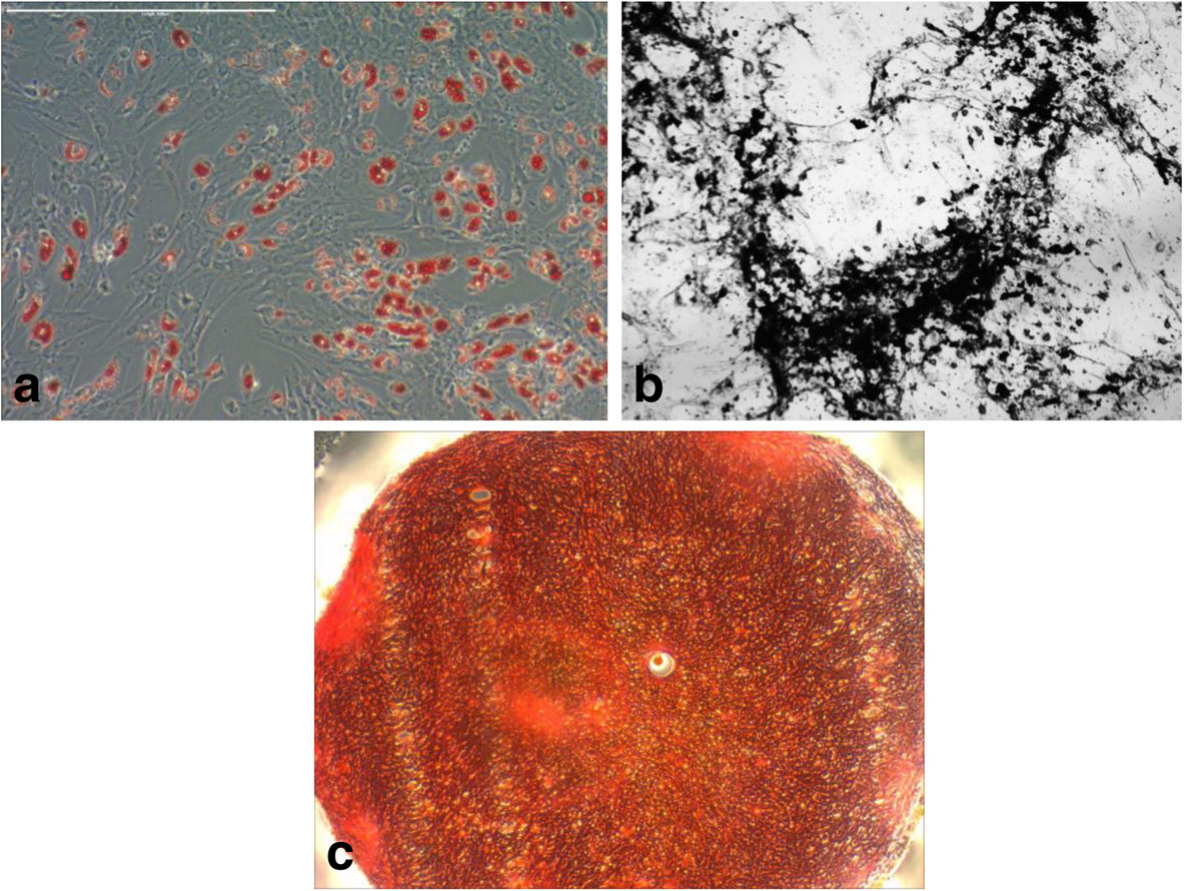
**a** Image of MSCSM (20X), 14 days after differentiation in adipocytes. Lipid droplets were detected (Oil red-O labeling). **b** Image of MSCSM (20X), 14 days after osteogenic differentiation, (staining in *Von Kossa*). **c** Image of MSCSM (20X), 21 days after differentiation in chondrocytes (staining in *Safranin O*)

The staining with Oil red marked the lipid droplets inside the cells on the adipogenic induction medium. The osteogenic differentiation was confirmed with the Von Kossa staining of the differentiated cells, evidencing the deposition of calcium formed during osteogenic differentiation. Control cultures did not exhibit differentiation. The chondrogenic potential was evaluated using the pellet culture system. Cells submitted to chondrogenic differentiation were stained with Safranin O evidencing the chondral extracellular matrix during cartilage differentiation (Fig. 2).

**Fig. 3:**
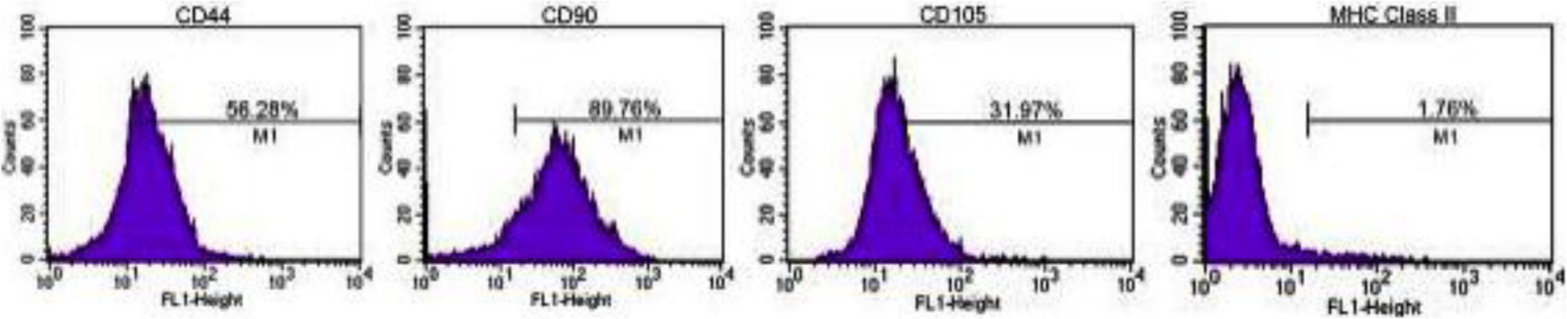
Immunophenotypic analysis of MSCs derived from equine synovial membrane. Histogram representing the flow cytometry performed on the MSC in the 1st passage using the following markers: CD44, CD90, CD105 and MHC Class II

Flow cytometric analysis revealed expression by CD90 and CD105 in all passages tested (1st to 3rd passages), determining the inter-species cross-reaction between mouse and equine, and between human and equine, respectively (Fig. 3). CD44 also reacted with MSC_SM_ in all passages. As expected, there was no MHC Class II marker reaction in MSC_SM_ (Table 1).

**Table 1:** Mean value of the expression of the markers used in MSC_SM_ surface analysis by flow cytometry in the 1st passage (P1) and 3rd passage (P3)

	P1	P3
CD44	55.3% ± 12.31	38.43% ± 6.12
CD90	92.49% ± 9.81	95.29% ± 5.19
CD105	90.25% ± 8.00	87.50% ± 11.01
MHC Class II	1.00% ±5.23	2.50% ± 1.50

The cell growth up to 80% confluence for the first pass (P1) occurred on average at 15 days, and the culture time between P1 and P3 occurred on average 30 days, probably due to the age of the donors, allowing faster growth.

**Fig. 4:**
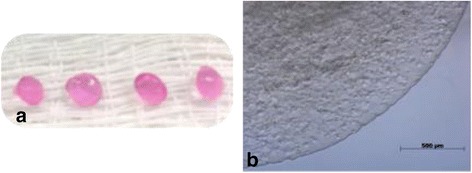
Mesenchymal stem cells derived from the equine synovial membrane inserted into 1.5% (*w*/*v*) alginate microcapsules with 21G needle. **a** Alginate microcapsules after gelatinization with 1000 μm diameter; **b** Phase contrast inverted microscopy of the cell culture in alginate hydrogel, it is observed numerous spherical and rounded structures that represent the high cellularity, objective of 20×

**Fig. 5:**
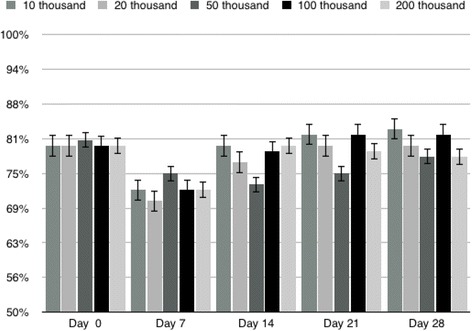
Proportion of mesenchymal stem cells derived from equine synovial membrane live up to 28 days after dissolution of the microcapsules in all groups (10 thousand, 20 thousand, 50 thousand, 100 thousand and 200 thousand cells)

In the present study, the microcapsules were made with 1.5% alginate solution, 21G needle and had a mean diameter of 1000 μm (Fig. 4). Thus, the use of 21G needle in the present study took into account the factors, seeking to minimize interference in cell viability and proliferation, maintaining cell viability at 80% and cell proliferation for 4 weeks, making the results as efficient as possible. When analyzing the number of live cells, it was not possible to observe statistical difference between the groups up to 28 days (Fig. 5), that is, all groups studied had the same behavior and growth curve, with live and dead cells in proportion Statistically the same over the evaluation time. On day 7 we observed a decrease in the number of live cells in all groups (with an increase in the proportion of dead cells). Thus, the initial decline in viability is expected, and in the present study occurred on the 7th day of evaluation, possibly due to the adaptation of the cells to scaffold.

As mentioned previously, in the following intervals of evaluation, the cells of the different groups maintained similar growth curve, showing expansion. The statistical evaluation shows that there was no lower growth in the groups with higher cell concentration, which could happen by competition for space or nutrients inside the microcapsule. With this, we can infer that it is possible to use different concentrations by microcapsules maintaining their viability up to 28 days.

Analyzing the results of the total number of living cells on different days and different concentrations (Table 2), we can observe that there was statistical difference between almost all groups when analyzed moment by moment. This difference can be explained by the disparity between the initial concentrations of each group. As mentioned above, the growth curve (percentage of live cells over the analyzed period) of each of the groups is similar. Thus, it is to be expected that the differences, as regards the total count of living cells, will be maintained. The exception occurred on day 14, where the groups of 20^4^ and 50^4^ cells had no statistical difference between themselves, as well as between the groups of 10^5^ and 20^5^ cells at the same time of evaluation. This fact probably occurred because of the proximity in the initial concentration of cells of these groups.

**Table 2:** Total number of mesenchymal stem cells derived from synovial membrane up to 28 days in all groups (10 thousand, 20 thousand, 50 thousand, 100 thousand and 200 thousand cells)

Moments groups	Day 0	Day 7	Day 14	Day 21	Day 28
10 thousand	10.000 ± 1,00 a A	19.000 ± 2,86 b A	46.665 ± 10,4 c A	57.500 ± 5,00 d A	63.333 ± 3,88 d A
20 thousand	20.000 ± 1,00 a B	12.666 ± 1,44 b B	23.333 ± 13,2 a A	18.889 ± 1,44 a B	20.555 ± 1,44 a B
50 thousand	50.000 ± 1,00 a C	55.833 ± 3,81 b C	33.333 ± 8,03 c A	36.666 ± 1,44 d C	37.500 ± 2,50 d C
100 thousand	100.000 ± 1,00 a D	40.000 ± 9,01 b D	85.000 ± 2,50c B	94.166 ± 3,82 a D	115.000 ± 5,00 d D
200 thousand	200.000 ± 1,00 a E	83.333 ± 6,30 b E	75.833 ± 29,8 c B	83.333 ± 27,5 b D	93.333 ± 27,5 b E

Different lowercase letters indicate differences between moments in the same group and different capital letters indicate differences between groups at the same time, statistical difference considered when the value of *p* < 0.05

When we observed the total number of live cells in the course of the intervals within each group, several differences were found indicating mainly the increase in the total number of living cells with the passage of time. The largest exception occurred with the group of 20^4^ cells, which practically did not show growth in the experimental period, showing the worst evaluation group. Over the 28 days, it was possible to observe that the groups of 10^4^, 10^5^ and 20^5^ cells had a higher number of total cells at the end of the experiment. However, only the 10^4^ and 10^5^ cell groups showed cell proliferation, with the total number of living cells larger at the end of the experiment period.

The results obtained in the present study allow to conclude that the group of 10^4^ can be used for intra articular application in equine, but several microcapsules would be necessary to reach the total number of MSCs required for the desired effect, and the articular volume is limiting. Already the group of 10^5^ cells maintained the similar growth curve, obtaining greater cell proliferation at 28 days. However, it is important to note that larger concentrations of microcapsules should be carefully evaluated so that there is no impairment of long-term cell viability after encapsulation. In the present work, for example, the concentration of 20^5^ cells had worse performance than that of 10^5^ cells, when we observed the total number of cells at the end of the experiment (28 days), since, comparing these two groups with higher concentration of cells, only 10^5^ ended the analysis period with more cells than it started. These observations indicate likely limit of cell concentration per capsule.

**Fig. 6:**
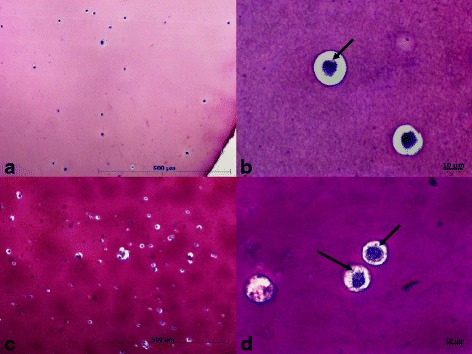
Optical microscopy of alginate microcapsules with mesenchymal stem cells derived from equine synovial membrane (MSC_SM_) at 7 and 21 days stained with toluidine blue, objective of 10 and 40×. **a** and **b** 7 days, rounded cell shape is observed, the arrows indicate the presence of gaps; **c** and **d** 21 days, more cells are observed, the arrows show the deposition of the proteoglycan matrix

Observation of alginate hydrogel cells at 7 days of culture by toluidine blue staining demonstrated low numbers of spherical cells with chondrocyte characteristics (Fig. 6a). At higher magnification (Fig. 6b) the formation of the pericellular matrix with gaps around the chondrocytes and retention of spherical morphology similar to that of native cartilaginous tissue was evidenced. During the 21 days of culture, the differentiation was more evident (Fig. 6c and d), with high cellularity compared to the culture at 7 days, indicating the differentiation and multiplication of these cells in the hydrogel, with pericellular and territorial matrix production.

## Discussion

Isolation of the equine MSC_SM_ was performed using a methodology previously described for humans [33] which SM from human patients with degenerative joint disease was collected and performed enzymatic digestion with collagenase enzyme to extract tissue cells and obtain shorter culture time. Similar results were observed in another study, where cells maintained the phenotypic characteristics up to passage 11 (P11) [34].

When human MSC_SM_ are cultured, it was observed that the primary culture time (P1) ranges from 20 to 25 days [33]. MSC_SM_ from healthy horses, aged from 2 to 10 years, obtained by the insertion method, had mean culture time to P3 of 68 days [35]. In studies using collagenase for cell isolation, as in the present research, cell expansion and confluence were observed in the second week of culture [18, 33, 36]. Researchers have related the age of donors as a relevant factor in culture time. In a study with rats, they observed that the cells had a decline in regenerative potential with the increase of donor’s age. In the present study, the animals were between 3 and 5 years old and could be considered as young animals, which favored cell proliferation. In the studies discussed above, animals of older ages were used were used, which may have lead to a slower cell growth with the need of longer culture time [37].

The encapsulation of the MSCs was performed in different ways, ranging from the concentration of the alginate solution to the gauge of the needle. 1.2% (*w*/*v*) alginate solution was used to encapsulate bovine chondrocytes using 22G needle [12]; 1.5% alginate solution and 21G needle [36]; 1.2% and 16G [38], 2% alginate solution in a few of 22 mm in diameter and 2 mm in height [18]. These variations (alginate concentration, capsule size, needle size used) may interfere with cell viability. A study using six different types of comercial alginate demostrated that type may influence chondrocyte culture upon finding variation in their pH, viscosity or n-gururonic acid-manuronic acid ratio [39]. Researchers confirmed that variations alginate concentration influence the cellular metabolism and diffusion of the necessary components of the culture medium, recommending the use of 1.5% (w/v) [30]. Needle diameter may interfere with cell viability, concluding that larger gauge (19G) needles for cell implantation reduce the loss of stress-induced viability compared to smaller (21G and 23G) needle gauges [40].

In addition to the above-mentioned variations, there are variations in cell concentration per microcapsule, described in the literature, varying from ten thousand cells [36] to sixty thousand cells per microcapsule [37]. In the treatment of joint lesions in horses, it is recommended to use 10 to 30 million cells to obtain an efficient clinical effect [41]. Thus, the amount of microcapsules to be applied at the joint is directly related to the concentration of cells in each microcapsule. The use of a larger number of cells by microcapsules makes the clinical application in equine possible, concentrating the number of cells in a smaller volume, and allowing the application of the number of cells necessary for therapeutic effect using approximately 100 microcapsules. As the volume of the target joints is usually limited, it becomes extremely useful to concentrate more cells / microcapsules in a way that makes the MSC_SM_ application technique in microcapsules viable in the clinical routine.

The cell death observed in the early stages, probably occurred due to the increase of the metabolic activity inside the microcapsules soon after the manufacture [42]. These same authors reported that in three-dimensional structures cell death occurs at the center and bottom of the scaffold, justifying the decline in viability in the first weeks after implantation. It has been observed a decline in cell viability 2 days after culture in 4 mm thick alginate [43].

Similar results to the present study were observed using 10^4^ cells per microcapsules, through maintenance and cell proliferation for up to 4 weeks. With similar methodology to the present study, using 10^4^ cells per microcapsules, was observed through maintenance and cell proliferation for up to 4 weeks [30]. Studies that performed the encapsulation of MSC from human bone marrow, at the concentration of sixty thousand cells per alginate microcapsule, reported that viability remained high (> 90%) over 4 weeks and the duration of culture in vitro had no effect on the cell viability [37]. By increasing the density per capsule, the number of microcapsules to be transplanted will be smaller, reducing the tissue damage produced by the transplant [44, 45].

Some characteristics were responsible for the selection of the alginate hydrogel in the present study: ability to solidify in the presence of calcium [12, 46], biocompatibility, injectable in animal models, it has chondroinducing properties to produce an environment similar to cartilage tissue and does not interfere with cell viability [47], which is characterized by the presence of cartilage, which has been shown to be responsible for the development of cartilage.

A number of studies have reported the potential of alginate stimulation in chondrogenic differentiation, stimulating both synovial membrane cells [18], and adipose tissue [48], human bone marrow [37], and bovine chondrocytes [12], but no research has evaluated the potential of equine cells, which makes the present work unprecedented. At 7 days of cultivation, has been described the same morphological finding when working with human bone marrow MSCs [37]. The low number of cells with chondrocyte characteristics found at this time can be explained by the hypothesis that the remaining cells remained undifferentiated, with the possibility of paracrine actions, with immunomodulatory and anti-inflammatory effects [49].

The staining with toluidine blue revealed the production of proteoglycans, the main component of this region. It has been obtained the same differentiation of MSCs from human adipose tissue in alginate beads at histological evaluation at 21 days [48]. The presence of toluidine blue stained gaps was described when using the three-dimensional culture with the alginate beads, caused by the stiffness of the matrix [19]. These same authors considered this coloration an advantageous method for the in vitro study of chondrocytes.

They It has been reported a significant increase of aggrecan and type II collagen levels, confirming the differentiation of cells into chondrocytes [18, 50]. These findings, together with the results of the present study, allow us to predict that alginate improves chondrogenic differentiation. In addition to chondrogenic differentiation improvement, it acts on cell maintenance, following the principles of three-dimensional scaffolding, keeping cells in an artificial, biodegradable matrix that can support the growth of cartilage for a few months, both for chondrocytes and matrix while chondrocytes and matrix [25, 47].

## Conclusion

The encapsulation technique used was easy to perform. The MSC_SM_ obtained good adaptation to the sodium alginate scaffold. All groups obtained the same proportion of living cells during the study period, but differed in the total number of living cells present. The concentration of 10^5^ was shown to be more efficient for the use of intra-articular application in horses, since it concentrates a larger number of cells since the articular volume is reduced. The hydrogel met the characteristics of the scaffold, maintaining the cells and stimulating the differentiation of the MSCs into chondrocytes. In vivo studies are needed to validate the therapeutic potential of the intra-articular application of the encapsulated MSC_SM_, but the results obtained to date are promising.

## Abbreviations

DMEM: Dulbecco’s Modified Eagle’s Medium
FBS: Fetal Bovine Serum
HA: Alginate hydrogel
HE: Hematoxylin and eosin
IM: Intramuscular injection
IV: Intavenous injection
MHC: Major histocompatibility complex
MSC: Mesenchymal stem cells
OA: Osteoarthritis
SM: Sinovial membrane
TB: Toluidine blue
