# Correction to: Culture of mesenchymal stem cells derived from equine synovial membrane in alginate hydrogel microcapsules

**DOI:** 10.1186/s13287-018-0999-6

**Published:** 2018-10-07

**Authors:** Vitor Hugo Santos, João Pedro Hübbe Pfeifer, Jaqueline Brandão de Souza, Betsabéia Heloisa Gentilha Milani, Rogério Antonio de Oliveira, Marjorie Golim Assis, Elenice Deffune, Andrei Moroz, Ana Liz Garcia Alves

**Affiliations:** 1 Department of Veterinary Surgery and Anesthesiology, University of Veterinary Medicine and Animal Science UNESP, District of Rubião Júnior, s / n, Botucatu, São Paulo, Brazil; 2 Departament of Statistics, Institute of Biosciences, UNESP, District of Rubião Júnior, s / n, Botucatu, SP Brazil; 3 Departament of Graduate Program in Research and Development, Medical Biotechnology, UNESP, Blood Centre Division, District of Rubião Júnior, s / n, Botucatu, SP Brazil; 4 Departament of Urology, Blood Centre Division - Laboratory of Cellular Engineering, University of Medicine, UNESP, District of Rubião Junior s / n, Botucatu, SP Brazil; 5 Departament of Bioprocesses and Biotechnology, FCFAR – UNESP, Rodovia Araraquara Jaú, KM 01, São Paulo, Brazil

## Correction

The original article [1] contained a minor error regarding the mean diameter of the alginate microcapsules described in relation to Fig. 4 in the Results section. The microcapsules had an actual mean diameter of 3000 μm instead of 1000 μm as mistakenly mentioned in the original article.

## References

[1] Santos VH (2018). Culture of mesenchymal stem cells derived from equine synovial membrane in alginate hydrogel microcapsules. Stem Cell Res Ther.

